# Secondhand effects of alcohol use among students in Vietnam

**DOI:** 10.3402/gha.v8.25848

**Published:** 2015-03-02

**Authors:** Pham Bich Diep, Ronald A. Knibbe, Kim Bao Giang, Nanne De Vries

**Affiliations:** 1 Institute for Preventive Medicine and Public Health, Hanoi Medical University, Vietnam; 2 Department of Health Promotion, CAPHRI School for Public Health and Primary Care, Maastricht University, Maastricht, The Netherlands

**Keywords:** students, secondhand effects, alcohol, non-bodily effect, bodily effects

## Abstract

**Background:**

In many countries worldwide, heavy drinking can cause harm not only to drinkers but also to those around them.

**Objective:**

To examine the prevalence and predictors of secondhand effects of alcohol use among students in Vietnam.

**Design:**

In this cross-sectional study, a multistage sampling strategy was used to select 6,011 students (from the first to final study year) of 12 universities/faculties in four provinces in Vietnam. During class, students filled in a questionnaire asking for demographic information, and about alcohol-related problems and details of secondhand effects of alcohol during the past year. Exploratory factor analysis of the secondhand effects indicated two factors: non-bodily harm and bodily harm. A logistic regression model was used to explore the association between predictors and non-bodily harm and bodily harm.

**Results:**

The prevalence of secondhand effects of alcohol is high among students in Vietnam: 77.5% had non-bodily effects and 34.2% had bodily effects. More than 37% of the population reported three to four non-bodily effects and more than 12% reported two to three bodily harms due to the drinking of others. However, most respondents who reported secondhand effects experienced these less than once per month. Factors most strongly associated with the yearly non-bodily harm were the weekly drinking habits of the people the respondents live with, and living in a smaller city; the factor most strongly associated with the yearly bodily harm was the respondent's own alcohol-related problems. Moreover, weekly drinking habits of the people the respondents live with, and respondent's own alcohol-related problems are strongly associated with the frequent experience of non-bodily and bodily effects of alcohol.

**Conclusions:**

In addition to dealing with alcohol-related harm of drinkers themselves, preventing secondhand effects should also be a major focus of prevention policy.

Alcohol abuse by students not only impacts the young drinkers themselves but often affects the people around them. As the secondhand effects of alcohol can contribute to the larger picture of alcohol-related harms, the extent of this problem needs to be established. The secondhand effects of alcohol can include, for example, that the drinking of others leads to interrupted sleep or study, being insulted, property damage, violence, and unwanted sexual advances. Research on the harm alcohol causes to others shows that the prevalence worldwide has increased. In an Australian study among a population aged 18–65 years, the youngest group (18–29 years) was most negatively affected by the alcohol use of others (1). Also, a study among US students in 1998 estimated that, among 18–24 year olds, 3,674 died from alcohol-related traffic deaths (2); moreover, in this age group, these deaths increased by 4% between 1998 and 2001 (3). The most common harms of secondhand effects are interruption of sleep (60% of the US students) (4); interruption of sleep or study (32.9% among Canadian students) (5); being insulted or humiliated (29% among the US students) (2); being pushed, hit, or assaulted (13.3 and 15% among the US and New Zealand students, respectively) (2, 6); as well as damaged property (15 and 20% among the US and the New Zealand students, respectively) (4, 6). According to Hingson et al. (2), about 600,000 students were hit or assaulted by other students who had been drinking. Sexual assault was also a serious secondhand effect of alcohol. Sexual abuse among students in the New Zealand study was 28% (6). A study among US students reported that 5% of females and 1.5% of college students had been the victim of sexual assault (7); and in Canada, 10% of students reported sexual harassment (5). The predictors of secondhand effects of alcohol among students were the place of domicile (i.e. living away from the family), the geographical region (5), and a high rate of binge drinking in the colleges (8).

However, all the above findings are based on research among students in developed countries. In developing countries, basic information on secondhand effects of alcohol is largely lacking. Therefore, among Vietnamese students in different provinces, this study examines the prevalence of different kinds of secondhand effects of alcohol, frequency, and predictors of these secondhand effects.

## Methods

### Setting

This cross-sectional study involved 6,011 students (from the first to final study year) of 12 universities/faculties (economics, medicine, and technology) of four provinces, which represent four different geographical areas in Vietnam: HN (the capital, a cultural and political city) in the North; HCM (modern and economic center) in the South; Hue (historical city) in the Center Coastal; and BMT (remote city) in the central highland. According to the Vietnamese Urban Classification, HN and HCM are the two central-level cities (the two largest cities); and BMT (belong to Dak Lak) and Hue (belong to Thua Thien Hue) are the provincial capital cities. In term of inhabitants, HN and HCM are the most crowded cities (6,936,900 and 7,818,800 inhabitants, respectively), whereas Dak Lak and Thua Thien Hue have far fewer people (1,827,800 and 1,123,800 inhabitants, respectively) (9).

### Sampling

A multistage sampling strategy was used with ‘city’ as the first stratum, ‘university’ as the second stratum, and ‘study year’ (from first to final study year) as the third stratum. Sample size was estimated using the World Health Organization formula for sample size (10), assuming the prevalence of secondhand effect of alcohol to be similar to that in developed countries [*p=*0.21 (4–6)] a relative precision of 0.1 and a significance level of 0.05). The required sample size was 1,446 for each city. Sample sizes for each city were rounded to 1,500, making a total sample size in four cites of 6,000. Because we collected data at three universities per city, 500 students were selected per university. Within each academic year, one or two classes (depending on the total number of students in the class) were randomly selected for this study to ensure that the sample size included 85–125 students per academic year. Depending on the specialty of the university, this requires 4, 5, or 6 years of study to graduate in economics, technology, and medicine, respectively. At the data collection time, all students in the selected classes were invited in the study. As a result, the number of students (from the first to final study year) from the provinces of HN, BMT, HCM, and Hue included in this survey were 1,557; 1,526; 1,445; and 1,483, respectively, yielding a total sample size of 6,011 students.

### Questionnaire

Based on the literature (2–6), we developed a questionnaire to measure secondhand effects of alcohol. First, the questionnaire was piloted among 30 students at three universities in Hanoi City to ensure that they understood the meaning of all the questions. The questionnaire covered: 1) general demographic information and respondents’ own alcohol consumption pattern; 2) the drinking frequency of the people the respondents live with; and 3) the secondhand effects of alcohol. Questionnaires were distributed in the class by the investigators and the students received instructions on how to fill in the questionnaires.

1. Demographic information comprised the name of the university, name of the city, sex, age, and living arrangements. To examine the respondents’ own alcohol consumption and their environment, the Vietnamese version of the Alcohol Use Disorder Identification Test (AUDIT) was included. The AUDIT consists of 10 questions with a total possible score of 40. Questions 1–8 can be scored from 0 to 4, and questions 9 and 10 can be scored 0, 2, or 4. In the present analysis, a cut-off score of 8 was used to identify alcohol problems among Vietnamese students (11). An AUDIT score of <8 implies an alcohol intake with a low risk of alcohol problems and a score of ≥8 implies alcohol problems.
2. Questions were asked about the frequency of drinking of the people the respondent lives with: ‘How often do the people you are living with drink alcohol?’ (1=sometimes per week; 2=sometimes per month; and 3=sometimes per year/never/living alone).
3. Seven questions were used to measure secondhand effects of alcohol: sleep disturbances; property damage; unwanted sexual advance (touching, staring); study disturbances (loss of concentration/place because of the noise from drinkers); being insulted/quarrel; being beaten/pushed/fighting/hitting; and being in a traffic crash/accident. These questions asked about the frequency of secondhand effects of alcohol during the previous 12 months on a five-point scale (1=daily or almost daily; 2=weekly; 3=monthly; 4=less than one per month; and 5=never). The seven variables were recoded in two ways: first, the respondent did or did not experience a secondhand effect – this is called the ‘yearly prevalence’ (5=0=never vs. 1, 2, 3, or 4 indicating one did experience the effect at least once in the past year); and second, the respondent did often (1, 2, 3=1 indicates effects ≥1 per month), or not often (4, 5=0 indicates <1 per month) experience a secondhand effect of alcohol – this is called the ‘monthly prevalence’.

### Ethics

This study was approved by the Biomedical Research Ethics Committee of School of Public Health in Hanoi, Vietnam.

### Data analysis

Analyses were performed with SPSS for Windows (version 20).

Descriptive statistics were used to estimate the prevalence and frequency of secondhand effects of alcohol.

We used principal component analysis with Varimax rotation to extract factors from the seven items pertaining to secondhand effects using the original scoring from 1 to 5. To decide what factors to retain we used the Kaiser's criteria (eigenvalue>1 rule), the cumulative percentage of variance extracted, and interpretability of the extracted factors. Individual loadings of 0.4 or greater were used in the factor designation. Extracted factors were examined and named based on an analysis of the items loading on each factor and the meaningful interpretation. Two factors emerged from the analysis, which we interpreted as bodily and non-bodily secondhand effects, respectively. Items were recoded to indicate whether respondents experienced (1) or did not experience (0) such effects. Subsequently, scale scores were computed by summing up the scores on each of the items constituting the two factors. Cronbach's alpha was used to estimate the internal consistency of the scale scores. The resulting two scale scores ranging from 0–4 (non-bodily secondhand effects) and 0–3 (bodily secondhand hand effects) were used in the logistic regression as binary variables (0/1) indicating whether or not a respondent indicated to have experienced last year, respectively, one or more bodily, or non-bodily secondhand effects. Logistics regression analysis was conducted to test which variables discriminated between: 1) students that did or did not experience secondhand effects of alcohol during the previous 12 months, and 2) once a month, or more often. Independent variables are sociodemographic variables (sex, age, type of living situation, region); respondents’ own alcohol consumption; and the drinking frequency of the people the respondent lives with.

All independent variables are controlled for in the logistic regression. Results are presented as odds ratios (OR) with 95% confidence intervals (95% CI).

## Results

Characteristics of the sample: Students equally come from four provinces (HN: 25.9%, HCM: 24%, BMT: 25.4%, and Hue: 24.7%). The mean age of the students is 20.6 (SD=1.8), and about half of the students (49.3%) are women. About 40% (40.7%) of the students still live with their parents or with relatives, a little bit more (44.8%) live in rented houses, and 14.8% of the students live on campus.

Exploratory analysis results: The Kaiser-Meyer-Olkin measure of sampling adequacy was 0.78 (*p*<0.000) indicating that the sample is large enough to detect the factors. Correlation among items ranked from 0.15 to 0.46. There was one item ‘property damage’ loaded on two factors (Table 1). Based on the factor loadings and meaningful interpretation, we decided to put this item in factor 1 ‘non-bodily effect’. Two factors emerged. Factor 1 covered a ‘non-bodily effect’ (including four items: sleep disturbances, property damage, study disturbances, and being insulted/quarrel) with Cronbach's alpha of 0.68. Factor 2 was interpreted as a ‘bodily effect’ (including three items: unwanted sexual advance, being beaten/fighting/pushed/hit, and having a traffic crash/accident) with Cronbach's alpha of 0.55.

**Table 1:** Summary of exploratory factor analysis results for secondhand effects of alcohol among 6,011 students

	Component	
	
Items	Factor 1: non-bodily effect	Factor 2: bodily effect
Sleep disturbances	**0.8**	0.0
Property damage	**0.4**	0.5
Unwanted sexual advance	0.0	**0.6**
Study disturbances	**0.8**	0.1
Being insulted/quarrel	**0.7**	0.4
Being beaten/fighting/pushed/hit	0.2	**0.7**
Having a traffic crash/accident	0.1	**0.7**
Eigenvalues	2.6	1.1
% of variance	37.8	15.8
Cronbach's alpha	0.68	0.55

Bartlett's test of sphericity is significance (p<.001).

Of the students, 20.4% reported no secondhand effects, 47.6% reported either bodily effects or non-bodily effects, and 32.1% reported both bodily and non-bodily effects (data not shown).

Table 2 shows that the prevalence of non-bodily secondhand effects of alcohol is higher than that of bodily secondhand effects. During the previous 12 months, 22.5% of the students reported to have experienced no secondhand non-bodily effects, 77.5% experienced non-bodily effects only, 65.8% reported to have experienced no secondhand bodily effects, and 34.2% of the students experienced bodily effects only. Non-bodily effects were reported most often, with 44.5% of the population reporting two to three non-bodily effects in the previous 12 months. Reports of bodily effects are less frequent, 21.8% reported one bodily effect in the previous 12 months. The prevalence of experiencing secondhand effects once a month or more often is much smaller than the prevalence of experiencing these effects during the previous 12 months (Table 2).

**Table 2:** Prevalence, number and frequency of secondhand effects of alcohol in the past 12 months among 6,011 students in Vietnam during 2012–2013

	Factor 1: non-bodily harms		Factor 2: bodily harms	
		
	n	%	n	%
Prevalence of secondhand effect of each factor	4,659	77.5	2,056	34.2
Prevalence of secondhand effect by different types:				
Sleep disturbances	3,560	59.2		
Property damage	1,363	22.7		
Study disturbances	3,565	59.3		
Being insulted/quarrel	2,901	48.3		
Unwanted sexual advance			505	8.4
Being beaten/fighting/push/hit			1,264	21.0
Traffic crash/accident			1,204	20.0
Number of secondhand effects of alcohol				
0 secondhand effects	1,352	22.5	3,955	65.8
1 secondhand effects	1,080	17.9	1,311	21.8
2 secondhand effects	1,334	22.2	573	9.5
3 secondhand effects	1,339	22.3	172	2.9
4 secondhand effects	906	15.1		
Number and frequency of secondhand effects of alcohol				
≥1 time per month	686	11.4	138	2.3
<1 time per month	5,325	88.6	5,873	97.7

Sample size varies slightly for each category because of missing values.

In Table 3, in the two first columns, the association between yearly prevalence of respectively non-bodily and bodily harm with the predictors is presented. In the two last columns the same is done for monthly prevalence of secondhand effects.

**Table 3:** Logistic regression of yearly respectively monthly prevalence of secondhand bodily and non-bodily effects

	Yearly prevalence of secondhand effects				Monthly prevalence of secondhand effects			
		
	Non-bodily secondhand effect		Bodily secondhand effect		Non-bodily secondhand effect		Bodily secondhand effect	
				
	OR	95% CI	OR	95% CI	OR	95% CI	OR	95% CI
Gender								
Female	1		1		1		1	
Male	0.8**	0.7–0.9	0.9	0.8–1.1	0.9	0.7–1.2	1.9*	1.1–3.2
Age	0.9*	0.9–1.0	0.9	0.9–1.0	0.9	0.9–1.0	0.9	0.9–1.1
Living arrangement								
Parents/family relatives	1		1		1		1	
Dormitory	1.4**	1.1–1.7	0.9	0.8–1.2	1.4*	1.0–1.9	1.3	0.7–2.5
Rented accommodation	1.7***	1.4–2.1	1.2*	1.0–1.4	1.2	0.9–1.5	1.5	0.9–2.4
Region								
Hanoi	1		1		1		1	
Hue	2.7***	2.2–3.5	2.0***	1.6–2.5	3.0***	2.2–4.0	2.4**	1.3–4.5
Hochiminh	1.5***	1.3–1.9	1.9***	1.6–2.3	2.0***	1.5–2.8	1.7	0.9–3.1
BuonMeThuat	3.2***	2.5–4.0	1.7***	1.4–2.1	2.5***	1.9–3.4	1.6	0.8–3.0
Drinking frequency of people the respondent lives with								
No drinking/sometimes in a year	1		1		1		1	
Monthly	1.6***	1.3–1.9	1.3**	1.1–1.5	2.1***	1.6–2.8	1.5	0.8–2.8
Weekly	2.3***	1.8–2.8	1.4***	1.2–1.7	3.7***	2.8–5.0	2.7**	1.5–5.1
Respondent's drinking behavior								
AUDIT score <8	1		1		1		1	
AUDIT score ≥8	2.0***	1.6–2.6	2.2***	1.9–2.6	2.0***	1.5–2.5	3.2***	2.0–5.0

OR: odds ratio adjusted for gender, age, living arrangement, region, drinking frequency of the people the respondent lives with, and the respondent's own alcohol problems; CI: confidence interval.

* *p*<0.05;

** *p*<0.01;

*** *p*<0.001.

Table 3 shows that studying in a smaller city, living with people who drink weekly and monthly, and students’ own alcohol problems are most strongly associated with yearly and monthly experience of bodily and non-bodily harms. For non-bodily harms, the highest ORs for yearly and monthly prevalence are for the geographical region (BMT: OR=3.2) and weekly drinking of the people the respondents’ live with (OR=3.7). For bodily harms, the highest ORs are for one's own drinking problem (OR=2.2 for yearly prevalence and 3.2 for monthly prevalence).

Other variables associated with significantly higher ORs of non-bodily secondhand effects of alcohol are being female (OR=yearly prevalence of 1 vs. 0.8), being of younger age (OR=yearly prevalence of 1 vs. 0.9), living in a dormitory (OR=yearly and monthly prevalence of 1.4 vs. 1), and living in rented accommodation (OR=yearly prevalence of 1.7 vs. 1).

Living in rented accommodation is associated with a higher likelihood of yearly experience of bodily secondhand effects (OR=1.2). Being male is associated with a higher OR of monthly bodily secondhand effects (OR=1.9).

## Discussion and conclusion

Secondhand effects of alcohol are common among students in Vietnam, and non-bodily effects are much more common than bodily effects. In this study, the prevalence of sleep and study disturbances is similar to that reported among American students (4) but higher than that among Canadian students (5); the prevalence of property damage is higher in our study than that reported in the United States and New Zealand (4, 6); the prevalence of being pushed/hit in our study is similar to that in New Zealand (6) but higher than that in the United States (2, 3); and the prevalence of unwanted sexual advances is lower than that found in both Canada (5) and New Zealand (6).

Of the three strongest associations of secondhand effects (region, living with people who drink weekly/monthly, and students’ own alcohol problems), the highest ORs for non-bodily harms were for region (for yearly prevalence) and the drinking frequency of those the respondent is living with (for monthly prevalence), whereas the highest ORs for yearly and monthly bodily harms were for one's own drinking problems. Being female, having a younger age, and living in a dormitory/rented accommodation are associated with a higher likelihood of yearly experience of non-bodily secondhand effect, whereas living in a dormitory is associated with a higher likelihood of monthly experience of non-bodily secondhand effects.

Living in smaller cities (BMT and Hue) was associated with a higher likelihood of having experienced secondhand effects of alcohol than living in bigger cities (HN and HCM). The explanation might be that students studying in bigger cities are more familiar with the noises and chaos of the big cities, so they might be less sensitive to non-bodily harm than students studying in smaller city. This finding is supported by other studies. An Australian study indicated that, compared to those living in metropolitan areas, people who live in a remote/regional area are nearly twice as likely to use health services due to harm inflicted by other drinkers (12). Moreover, people living in smaller cities might drink more alcohol than those in larger cities and may experience a higher risk of harm from other drinkers. Another study conducted in a rural area of Vietnam supports this explanation; the latter study indicated that men in the highland area (less-densely populated) more commonly reported alcohol use and related problems compared to those in lowland areas (13). Also, a study in Australia reported that rural students drink more, are more likely to drink to get drunk, and therefore suffer greater harms (14). Another explanation is the availability of home-brewed alcohol at low prices in rural areas. A study in England reported that the availability of cheap alcohol is one of the causes for alcohol-related harm to others (15).

Students’ own alcohol-related problems are most strongly associated with the monthly prevalence of bodily effects; this finding is supported by many studies among students worldwide. Physical fighting, sexual assault, and injuries are associated with alcohol consumption among students (16, 17). Additionally, young binge drinkers frequently cause harm to others, as also reported in the United Kingdom (15). An association also exists between physical assault and drinking patterns of the victim (18).

Male students are less likely to report non-bodily effects but are more likely to experience bodily effects of alcohol. Sex differences in experience related to harm from others are also reported by others (19, 20). One explanation for this is that, because men may be more familiar with the noise/chaos or other types of non-bodily effects, they may be less sensitive to non-bodily harm than female students. Additionally, Pham et al. (21) show that Vietnamese male students drink more and are about 14 times more likely to have alcohol problems than female students (21). Hence, the male students’ own alcohol-related problems might cause the bodily effects of alcohol for themselves and others.

Additionally, we found that students living together with weekly drinkers are more likely to experience both bodily and non-bodily effects. A non-binge drinking or abstinent student living on a campus with students with a high frequency of binge drinking is twice as likely to be assaulted and three times more likely to have their sleep and study interrupted than those living in other campuses (22). A study in the United States also showed that residents of neighborhoods near a school with frequent binge drinking reported that they are more likely to be disrupted by noise, damaged property, and police presence (4).

The present findings provide evidence that alcohol-related harm to others is common among students in Vietnam, which suggests that both the drinkers and victims require attention. Managers at the community level should recognize the importance of alcohol prevention programs by developing projects in the local setting that promote health for the community. Authorities should aim to strengthen the activities of primary healthcare for those who are victims of drinkers. Additionally, alcohol policy needs to focus on reducing harmful drinking by implementing sales restrictions on alcohol for intoxicated persons. Government should set a minimum price unit for alcohol, especially for home-brewed alcohol. There is evidence that hazardous drinking, and serious alcohol use and its consequences among all age groups, can be reduced by increasing the price of alcohol (23). Furthermore, increasing the price and reducing the availability of alcohol helps to reduce alcohol use among young people (24).

In conclusion, this study provides insight into alcohol-related harms to others among students in Vietnam. The high prevalence of secondhand effects of harms among these students reveals the detrimental effect of harmful drinking in many areas in Vietnam, especially in the smaller cities. These data indicate that, apart from preventing harm among drinkers, alcohol prevention policy also needs to focus on preventing drinkers from harming others.

### Study limitations

This is the first study to examine secondhand effects of alcohol use among students in Vietnam. The study provides initial findings on the prevalence, frequency, and predictors of the possible types of secondhand effects of alcohol among Vietnamese students. Because of its cross-sectional design, no causal link can be drawn between the relationship with alcohol use. Also, as the responses are self-reported, we cannot exclude the possibility of some response bias.

Additional studies are needed to further examine the effects of alcohol use in Vietnam.

## Authors' contributions

Pham Bich Diep, Ronald A. Knibbe, and Nanne De Vries designed the study. Pham Bich Diep and Kim Bao Giang managed to collect the data and analyzed the data. Pham Bich Diep wrote the first draft with assistance from Ronald A. Knibbe, Nanne De Vries and Kim Bao Giang. All authors contribute ideas to improve the subsequent drafts and have approved the final manuscript.
